# Herpes Simplex Virus Type 1 Infection of Human Periodontal Ligament

**DOI:** 10.3390/ijms25158466

**Published:** 2024-08-02

**Authors:** Morgane Ortis, Marlène Chevalier, Charles-Vivien Olivieri, Sébastien Vitale, Adrien Paul, Lilit Tonoyan, Alain Doglio, Robert Marsault

**Affiliations:** 1 Laboratoire MICORALIS, Faculté de Chirurgie Dentaire, Université Côte d’Azur, 5, Rue du 22ème BCA, 06300 Nice, France; morgane.ortis@univ-cotedazur.fr (M.O.); marlene.chevalier@univ-cotedazur.fr (M.C.); charlesolivieri@sfr.fr (C.-V.O.); dradrienpaul@gmail.com (A.P.); lilit.tonoyan@univ-cotedazur.fr (L.T.); robert.marsault@univ-cotedazur.fr (R.M.); 2 Laboratoire de Virologie, Centre Hospitalier Universitaire de Nice, 06003 Nice, France; vitale.s@chu-nice.fr; 3 Unité de Thérapie Cellulaire et Génique (UTCG), Centre Hospitalier Universitaire de Nice, 06003 Nice, France

**Keywords:** periodontal ligament, viral infection, HSV-1, immune response, periodontal inflammation, oral diseases

## Abstract

The periodontal ligament (PDL) is a complex connective tissue that connects the tooth root to the dental alveolar bone and plays crucial mechanical roles. PDL also exhibits regenerative roles and regulatory functions to maintain periodontium integrity and homeostasis. While PDL exposure to oral microbial pathogens is common, virtually nothing is known regarding viral infections of PDL. In particular, human herpes simplex virus type 1 (HSV-1) persistently infects the oral cavity through infections of the oral epithelium, connective tissue and neurons. While the oral spread of HSV-1 is generally asymptomatic, this virus has also been implicated in various oral pathologies. In this study, using a primary cell model derived from PDL (PDL cells), and whole surgical fragments of PDL, we provide evidence supporting the efficient infection of PDL by HSV-1 and the promotion of cytopathic effects. Infection of PDL by HSV-1 was also associated with an acute innate inflammatory response, as illustrated by the production of antiviral interferons and pro-inflammatory cytokines. Furthermore, this inflammatory response to HSV-1 was exacerbated in the presence of bacterial-derived products, such as peptidoglycans. This work therefore highlights the ability of HSV-1 to infect mesenchymal cells from PDL, suggesting that PDL may serve as a viral reservoir for the periodontal spread of HSV-1. Moreover, this raises questions about HSV-1 oral pathogenesis, as HSV-1-associated cytopathic and inflammatory effects may contribute to profound alterations of PDL integrity and functioning.

## 1. Introduction

The periodontal ligament (PDL) is a unique thin connective tissue that covers the root of the tooth between the dental alveolar bone and the tooth cementum. The immediate function of the PDL is to attach the tooth to the alveolar bone through a dense network of fibers that allow withstanding the forces of mastication. This ligament tissue has a complex cellular content, made up of fibroblast-like cells, osteoblasts, osteoclasts, epithelial cell rests of Malassez, cementoblasts and odontoclasts, and exhibits highly structured microstructures, such as networks of blood vessels and sensory nerve endings [1,2,3]. In addition, PDL also contains mesenchymal stem cells (MSCs) able to differentiate into osteoblasts, cementoblasts and fibroblasts, allowing for periodontium regeneration and tissue repair [4,5]. Beyond its mechanical and regenerative roles, PDL also exhibits immune regulatory functions, eliciting a regulatory immune response to face acute inflammation triggered by periodontal pathogens [6,7,8,9]. Particularly, PDL cells have been shown to produce various cytokines and chemokines in response to different inflammatory stimuli, indicating that PDL cells are fibroblast-like cells able to act as immune cells [10].

While the oral cavity harbors a wide variety of pathogenic viruses, virtually nothing is known about the interplay between PDL and viral infections. Nevertheless, PDL exhibits distinct features that may facilitate viral infections, such as its cellular diversity and a vascular network intimately connected to tissue structures. Particularly, the human herpes simplex virus type-1 (HSV-1) is a common oral virus widely distributed in the human population [11]. Over 70% of the population shed HSV-1 asymptomatically in the oral cavity at least once a month, with many individuals appearing to shed oral HSV-1 more than 6 times per month [12]. Oral HSV-1 shedding can eventually result in a mild disease, such as herpes labialis, the vesicular lesions on or near the lips that are commonly known as cold sores [13]. HSV-1 is also capable of causing much more serious illnesses, including herpes stromal keratitis, herpes encephalitis and disseminated neonatal infections [14,15]. Recent evidence also suggests a potential contribution of HSV-1 to Alzheimer’s disease [16].

Over the past decade, several studies have highlighted the likely role of various human herpesviruses (HHVs), including Epstein–Barr virus (EBV), cytomegalovirus and HSV-1, in the pathogenesis of periodontitis [17,18]. Recent meta-analysis and examination survey based on large cohorts of patients revealed that HSV-1 was significantly associated with periodontitis, and notably with severe periodontitis [19,20]. While infections of epithelial cells and plasma cells have been proposed to support EBV spread in periodontal lesions [21,22,23] the mechanisms supporting HSV-1 periodontal pathogenesis are still elusive. It is well established that HSV-1 primarily exhibits tropism for epithelial cells and fibroblasts, and infects skin and connective dermal tissues. Subsequently, it infects the termini of neurons and travels in a retrograde manner to the neuronal cell body, where it remains in a latent state until reactivated by different stimuli [24,25]. Here, we explore the hypothesis that HSV-1 may infect mesenchymal cells originating from PDL, examining the possibility that the PDL may serve as a target tissue capable of sustaining HSV-1 infection in the periodontium.

## 2. Results

### 2.1. Implementation of a PDL-Cell Model

**Figure 1:**
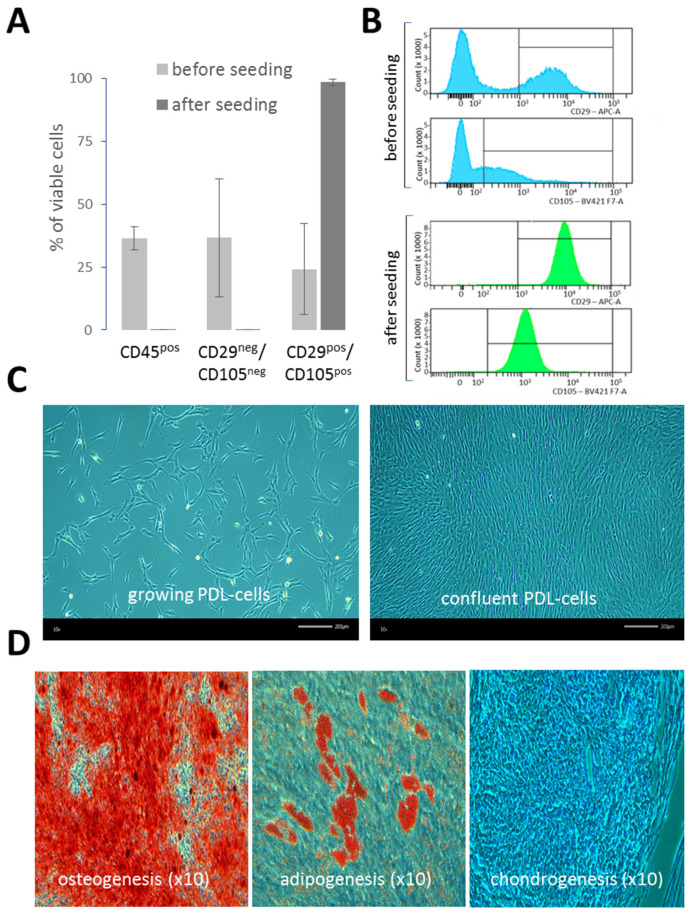
Characterization of PDL cells. (**A**) FACS analysis of PDL single-cell suspensions and PDL cells in cultures. Hematopoietic cells were identified by CD45, and mesenchymal cells by CD29 and CD105 in the CD45^negative^ population. PDLs from 6 donors before seeding and cultured cells from 4 PDLs at passages P1 to P7 (19 samples total) after seeding were analyzed. Bars represent the mean % of viable cell populations with standard deviation. (**B**) Representative FACS histograms showing CD105 and CD29 expression in CD45^negative^ PDL cell populations before (blue) and after (green) seeding. (**C**) Fibroblast-like morphology of growing and confluent primary PDL cells culture. (**D**) Osteogenic (**left**), adipogenic (**center**) and chondrogenic (**right**) differentiation potential of PDL cells. Images (×10) show calcium deposition and mineralization in osteocytes (alizarin red S staining), intracellular lipid droplets in adipocytes (oil red O staining) and micromass culture of chondrocytes (alcian blue staining).

Single-cell suspensions of PDL (n = 6) were extemporaneously characterized by flow cytometry. Overall, before seeding, FACS-analysis of single-cell suspensions of PDL revealed similar cell viability across donors (80% ± 4.8). However, there was notable variability in the frequencies of different cell populations, reflecting the inherent differences in the initial single-cell preparations from the crude tissue samples (n = 6). The PDL hematopoietic cell content (CD45) was 36.6% ± 19.1 and the mesenchymal cell content (CD29 and CD105) was 24.2% ± 18 (Figure 1A). Apart from hematopoietic and mesenchymal cells, PDL single-cell suspensions also contained other cell types (CD45^neg^ CD29^neg^ CD105^neg^) at 36.7% ± 23.4, which were not further characterized in this study. After the seeding of PDL single-cell suspensions in cell culture flasks, the phenotype of PDL cells was monitored from early (P1) to late cell passages (P7) (in a total of n = 19 samples from 4 different PDLs). The analysis revealed a highly homogeneous and stable mesenchymal cell phenotype, which was present very early after seeding (P1), predominantly comprised of cells expressing both CD29 and CD105 (98.1% ± 1.2). Of note (Figure 1B), CD105 exhibited a wide range of intensities in PDL cells before seeding, but after seeding, its expression became very uniform. In contrast, no major change was observed regarding CD29 expression in PDL cells before and after seeding. As illustrated in Figure 1C, cultured PDL cells exhibited mostly typical fibroblast-like shapes. In addition, PDL cells cultured in specific cell differentiation media were able to differentiate into osteogenic cells, adipocytes and chondrocytes (Figure 1D), confirming the presence of pluripotent MSCs in cultured PDL cells and their ability to differentiate in vitro. Each batch of PDL cells was maintained in culture over seven passages without a loss of growth capacity.

### 2.2. HSV-1 Infection of PDL-Cells

**Figure 2:**
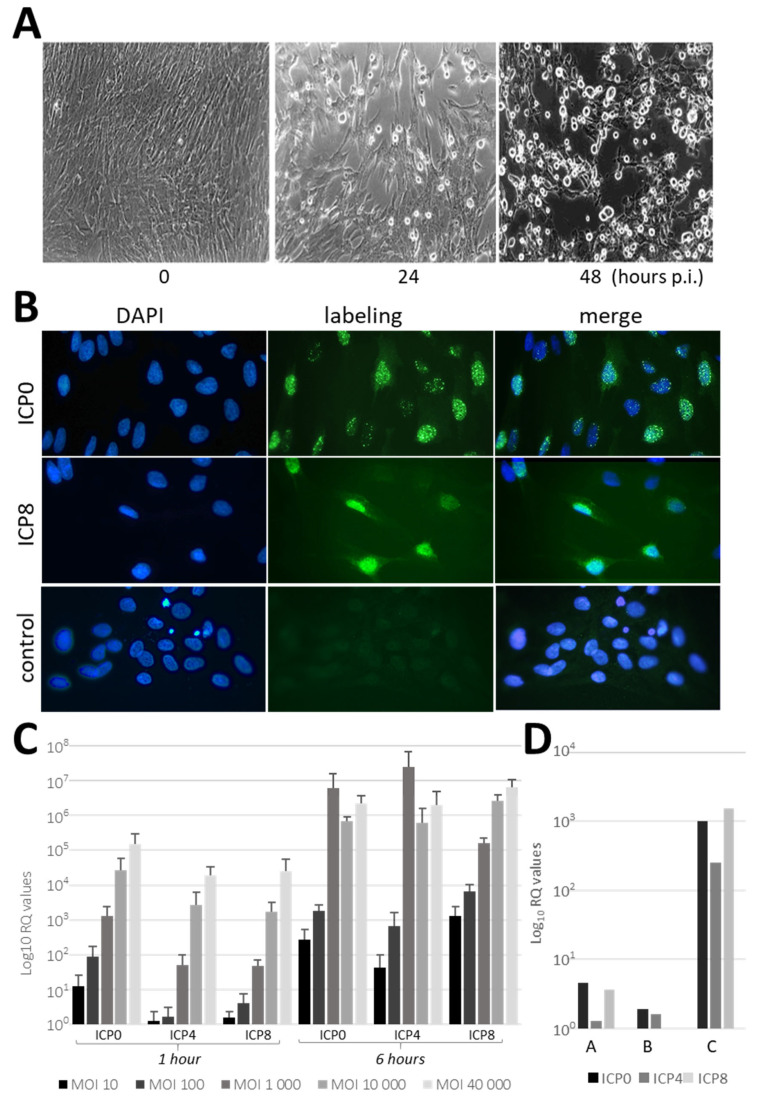
HSV-1 infection of PDL cells. (**A**) Morphological changes in PDL cells 24 and 48 h post-infection (p.i.) with HSV-1 (MOI 100), showing ballooning and detachment of dead cells (10× magnification). (**B**) Visualization of HSV-1 infection in PDL cells by immunofluorescent staining (green) of immediate-early (ICP0) and early (ICP8) viral proteins (10× magnification). Cells were infected with HSV-1 (MOI 100), fixed at 1 h p.i. (ICP0) and 24 h p.i. (ICP8). Control: irrelevant mouse IgG1; nuclei stained with DAPI (blue). Panels (**A**,**B**) are representative of 6 independent infections. (**C**) RT-qPCR of PDL cells infected with HSV-1 at various MOIs (10 to 40,000), measuring immediate-early (ICP0, ICP4) and early (ICP8) transcripts at 1 and 6 h p.i. Bars show mean expression values; error bars indicate standard deviations (n = 3). Viral titer-dependent (MOI) response to HSV-1 infection in PDL cells was determined using repeated measures ANOVA. The statistical significance and P-values of the comparisons are presented in Table S1 to avoid overcrowding the graph. (**D**) RT-qPCR of PDL fragments exposed to HSV-1 (4 × 10^7^ TCID50/mL) for 4 h, showing Log_10_ fold changes in ICP0, ICP4 and ICP8 transcripts from 3 donors (PDLs A, B and C). Baseline in (**C**) from mock-infected cells; in (**D**) from uninfected fragments of the same donors. Gene expression levels were calculated using the 2^−ΔΔCT^ method and normalized to GAPDH as a reference gene.

To determine whether PDL cells were permissive to HSV-1 infection, we performed in vitro infection studies on cultured PDL cells and PDL fragments (Figure 2). Infection of cultured PDL cells induced marked morphological changes, which were observed 24 and 48 h after the viral challenge (Figure 2A). These morphological changes, characterized by ballooning and subsequent detachment of the dead cells, highlighted the cytolytic activity of the virus in PDL cells.

The effectiveness of HSV-1 infection of PDL cells was demonstrated through the detection of the immediate (ICP0) and the early (ICP8) viral proteins by immunofluorescent staining (IF; Figure 2B). ICP0 was detected in cell nuclei within 1 h post-infection (p.i.) and ICP8 was abundantly accumulated in cell nuclei a few hours later (24 h). These results showed that there was no delay in the onset of HSV-1 infection in PDL cells. HSV-1 infection was also confirmed by RT-qPCR experiments to monitor viral gene expression in PDL. ICP0, ICP4 and ICP8 viral transcripts were expressed soon after infection of cultured PDL cells (1 h p.i.), reaching high levels of expression at 6 h depending on the viral titer (Figure 2C). This highlights the dynamic nature of HSV-1 infection in PDL cells. Higher MOIs resulted in a more rapid expression of the immediate-early gene ICP0 by 1 h p.i. compared to lower MOIs, while ICP4 and the early gene ICP8 peaked more prominently at 6 h p.i. By 6 h p.i., there was a comparable expression among ICP0, ICP4 and ICP8, and from an MOI of 10,000 onwards, differences between higher MOIs diminished. These findings illustrate a dose-dependent effect of HSV-1 infection on viral gene expression kinetics in PDL cells, with higher MOIs leading to earlier and more pronounced expression of immediate-early genes and a shift towards more balanced expression levels of early genes at later time points.

We then investigated the permissiveness of whole fragments of PDL tissues to HSV-1. PDL fragments (n = 3, from donors designated A, B and C) were placed in a medium containing cell-free virus for 4 h, and HSV-1 infection was monitored by the expression of viral transcripts (Figure 2D). An increase in ICP0, ICP4 and ICP8 expression was observed in PDL fragments following HSV-1 exposure. However, the levels of viral infection differed significantly between donors, with pronounced infection observed in PDL fragments from donor C and significant, but much lower, infection in PDL fragments from donors A and B.

### 2.3. Innate Antiviral Response in HSV-1 Infected PDL Exposed to Bacterial Products

**Figure 3:**
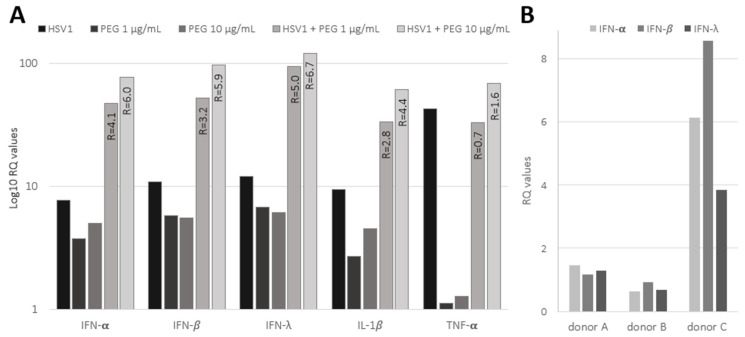
Innate immune response in HSV-1-infected PDL exposed to bacterial peptidoglycan (PEG). (**A**) Expression of interferons (IFN-α, IFN-β, IFN-λ) and cytokines (IL1-β, TNF-α) in cultured PDL cells after 24 h of HSV-1 (MOI 100) exposure with or without PEG. Bars show Log_10_ fold changes in gene expression. The synergy between PEG and HSV-1 was assessed by calculating the ratio (R, shown inside the bars) of the sum of Log_10_ RQ values for the combined condition (HSV-1 + PEG) to the sum of individual conditions. R > 1 indicates synergy, R ≈ 1 additive effect, and R < 1 antagonism. (**B**) Expression of interferons (IFN-α, IFN-β, IFN-λ) in PDL fragments from 3 donors (PDLs A, B, C) after 4 h of HSV-1 (4 × 10^7^ TCID50/mL) exposure. Bars represent fold changes in gene expression. (**A**,**B**) Gene expression was calculated using the 2^−ΔΔCT^ method, with GAPDH as the reference gene and mock condition as the reference sample.

The expressions of pro-inflammatory cytokines (IL-1β, TNF-α) and markers of the innate antiviral response (IFN-α, IFN-β and IFN-λ) were investigated by RT-qPCR in PDL cells infected with HSV-1 (MOI 100, 24 h) and exposed to bacterial-derived products, such as peptidoglycan (PEG; Figure 3A). The basal levels of IL-1β and TNF-α exhibited a significant increase (9.5- and 43-fold increase, respectively), indicating efficient induction of pro-inflammatory response in PDL cells upon HSV-1 infection. Viral infection was also associated with a marked increase in the antiviral innate response, evidenced by about a 10-fold increase in IFN-α, IFN-β and IFN-λ expression. Conversely, PEG, tested at concentrations of 1 and 10 μg/mL, exhibited a slightly weaker effect on cytokines and IFN response compared to HSV-1. Notably, TNF-α weakly responded to PEG. However, the combination of PEG and HSV-1 infection resulted in a remarkable synergistic effect, significantly boosting the induction of the innate immune response. As indicated in Figure 3A, the ratio between the effect of the HSV-1 and PEG combination and the sum of their individual contribution showed a marked synergistic upregulation of IFN-α, IFN-β, IFN-λ and IL-1β expression. In contrast, no or very low synergism was observed for TNF-α, its stimulation was mainly attributed to the virus.

Moreover, the IFN antiviral response (IFN-α, IFN-β and IFN-λ) was also analyzed in PDL fragments infected with HSV-1 (Figure 3B). The induction of IFNs remained weak at 4 h p.i. in A and B PDLs; however, strong induction was observed in C PDL, which displayed the highest level of viral expression, as shown in Figure 2D. These results suggested that the IFN response was related to the level of viral infection in PDL fragments and that viral spread in the PDL was able to promote a rapid and marked inflammatory response.

## 3. Discussion

In this study, we established a primary cell culture model representative of mesenchymal PDL cells. Although this culture system oversimplifies the PDL tissue complexity, it provides a reliable and suitable model for HSV-1 infection of PDL considering that, apart from neurons, the main cell tropism of HSV-1 are stromal cells. One of the main advantages of this model is its ability to maintain a homogeneous phenotype of PDL-derived mesenchymal cells over the long term in cell culture while preserving their capacity for differentiation. Indeed, more than 99% of cultured PDL cells met all the required criteria to be identified as mesenchymal cells with a fibroblast phenotype, in particular, plastic adhesion, expression of mesenchymal markers (CD105, CD29, vimentin), lack of hematopoietic markers (CD45) and the ability to differentiate into osteoblasts, adipocytes and chondrocytes [26]. These observations thus clearly established that HSV-1 infects mesenchymal fibroblasts derived from PDL. However, more exhaustive experiments would be needed to determine whether there are minor phenotypic differences within this fibroblast population and whether these subtypes could present differences in sensitivity to HSV-1. Using this system, we brought the first evidence that PDL may support HSV-1 infection. HSV-1 was shown to quickly spread in PDL cell cultures promoting profound morphological changes and cytopathic effects within 24 h. This model proves to be highly effective for supporting HSV-1 infection in vitro, demonstrating the capability to produce large amounts of infectious HSV-1, at levels comparable to or even higher than those produced by Vero cells.

Furthermore, HSV-1 was also able to infect PDL fragments revealing viral permissiveness in the whole tissue. Previous studies have shown that HSV-1 invasion in oral mucosa can be restricted by mechanical barriers [27]; however, we did not observe any restriction and delay regarding HSV-1 infection in cultured PDL cells and PDL fragments, since immediate-early (ICP0 and ICP4) and early viral transcripts (ICP8) were expressed as soon as 1 h after viral exposure. However, we assume that our experiments may have some limitations, considering microlesions caused by scraping and PDL detachment could have favored the viral entry and diffusion into the tissue. In whole tissue, the level of HSV-1 infection greatly varied between samples, which may reflect the differences in tissue organization and cell content among PDL samples collected by dissection from different individuals. Additionally, the presence of different factors may regulate HSV-1 infection, as suggested by previous studies demonstrating that salivary factors can modulate the infection of AG09319 gingival fibroblasts [28].

Overall, while our study benefited from using simplified and reliable in vitro models of PDL, this approach also presents a significant limitation. Although in vitro models offer controlled experimental conditions suitable for mechanistic studies, they lack the complexity of in vivo infections where interactions with immune cells are crucial. The absence of immune cells in our experimental setup restricts our understanding of how HSV-1 interacts with the host immune system during periodontal infections. To address this limitation, future investigations could explore more sophisticated models that better simulate the oral environment. Several potential in vivo models could be considered for studying HSV-1 infection in PDL cells and its interactions within the oral environment. Organotypic culture models of human oral tissues provide a more realistic three-dimensional environment to examine HSV-1 entry, replication and spread. Mouse models provide a controlled setting to explore HSV-1 pathogenesis and immune responses in the oral cavity. Humanized mouse models, involving engrafting mice with human immune cells or tissues, enhance translational relevance by allowing the study of HSV-1 infection in the presence of human immune components. Finally, clinical studies involving HSV-1-infected individuals with periodontal diseases will provide valuable clinical data on viral load, persistence and immune responses directly in affected tissues. Our ongoing research includes one such clinical study to further explore these aspects.

Innate immunity is crucial for an effective host defense against pathogenic microorganisms in periodontal tissues. PDL cells have been shown to synthesize immunomodulatory cytokines that are believed to influence the local response to infections [6,7,8,9]. We thus analyzed the innate immune response of the PDL exposed to HSV-1 and concluded that viral infection promoted an acute pro-inflammatory immune response in both cultured PDL cells and PDL fragments with a significant increase in TNF-α and IL-1β and induction of innate antiviral cytokines such as the type I interferons (IFN-α and IFN-β) and type III interferon (IFN-λ). The level of IFN induction in response to HSV-1 appeared lower in HSV-1-infected PDL fragments than in cultured PDL cells. However, this difference may be explained by the duration of viral exposure, which was adjusted to each experimental system (i.e., 4 h versus 24 h, respectively). Moreover, the innate response of PDL cells was shown to be over-stimulated when cells were infected with the virus in the presence of bacterial-derived products such as PEG. Although future studies will be needed to confirm synergistic effects through in-depth quantitative methods, the initial results presented here show that the combination of HSV-1 and PEG strongly enhances the inflammatory response of PDL cells. It has been proposed that the exacerbation of periodontal pathogenesis may involve synergistic interactions between periodontal bacterial dysbiosis and viral infections. Our results thus highlight the possibility that PDL may contribute to acute periodontal inflammation when exposed to different pathogen-associated molecular patterns from both bacteria and viruses.

In conclusion, the present study provides evidence to support that PDL may represent a reservoir for HSV-1 spread not only in the periodontium but also beyond in the oral cavity. HSV-1 shedding is common in the oral cavity, and its abundance in inflammatory periodontal sites has been reported. Furthermore, PDL is densely innervated and vascularized tissue, with HSV-1 infections typically occurring via the neural route and possibly also via the hematogenous route [29]. These factors collectively suggest a high probability of HSV-1 spreading in PDL in vivo, although further investigations are needed for confirmation. However, the presence of HSV-1 in PDL may promote severe immune dysfunction and significant alterations in PDL organization due to its cytopathogenic effect. This assumption opens the door to further investigations into the infection of periodontal tissues by HHVs and highlights the possible need for antiviral therapy. This therapy should be based on the administration of anti-HHV drugs to patients suffering from periodontal diseases, notably periodontitis.

## 4. Materials and Methods

### 4.1. Preparation of PDL Fragments and PDL Single-Cell Suspensions

PDLs were collected from healthy individuals undergoing surgery for wisdom teeth removal. In total, PDLs from 9 healthy donors were used in this study, 6 for flow cytometry analysis and PDL cell seeding, and 3 for ex vivo HSV-1 infection of PDL fragments (donors A, B and C). This study is classified as non-interventional research involving acts devoid of risks for the patients (category 3 in the context of the French ‘Jardé Law’). Informed consent was obtained from all subjects involved in the study to inform him/her of his/her right to oppose the use of his/her specimens and data for research purposes (authorized biomedical collection N°DC-2022–5040, French Research Ministry). 

Extracted teeth were immersed in phosphate-buffered saline (PBS) containing antibiotics (100 U/mL penicillin and 100 μg/mL streptomycin) and antifungals (2.5 µg/mL amphotericin B and 0.5 µg/mL caspofungin) and kept at 4 °C for less than 24 h. After 3 successive washings in PBS, rare pieces of gingival tissue still attached to the tooth were carefully removed by dissection. PDL tissues were then collected through scalpel-scraping of the mid-third of the root surface, washed with PBS and chopped into small pieces of tissue a few millimeters in size (PDL fragments). A single-cell suspension of PDL cells was obtained after digestion of PDL fragments with 3 mg/mL type I collagenase and 4 mg/mL dispase II (Life Science, Sunnyvale, CA, USA) for 30 to 40 min at 37 °C with vigorous shaking every 10 min. The digested PDL fragments were then passed through a 70 μm cell strainer (BD Falcon, Franklin Lakes, NJ, USA) and centrifuged at 400× *g* for 5 min. The cells were resuspended in α-MEM supplemented with 0.292 mg/mL L-Glutamine and 20% heat-inactivated fetal calf serum (FCS), and then counted using a Malassez-counting chamber.

### 4.2. PDL Cell Culture and Differentiation

PDL single-cell suspensions were plated into 6-well plates (Falcon, Dutscher, Bernolsheim, France) containing α-MEM supplemented with 20% heat-inactivated FCS, 100 U/mL penicillin, 100 μg/mL streptomycin, 2.5 µg/mL amphotericin B and 0.5 µg/mL caspofungin and then incubated at 37 °C with 5% CO_2_ in a humidified atmosphere. Cells were typically plated at a density of 3 × 10^5^ cells per well and dissociated with 0.25% Trypsin/0.02% EDTA upon reaching 80–90% confluency. The cells were subcultured in α-MEM supplemented with 10% heat-inactivated FCS, 100 U/mL penicillin and 100 μg/mL streptomycin, defined as a complete culture medium (CCM). Cells from passages P1 to P7 were used for experiments. For osteogenic and adipogenic cell differentiation, PDL cells at 80% confluency were incubated for 4 weeks, either in osteogenic medium (CCM supplemented with 50 µg/mL L-ascorbic acid 2-phosphate, 5 mM sodium β-glycerophosphate, 100 nM dexamethasone) or in adipogenic medium (CCM supplemented with 10 µg/mL insulin (Gibco, Grand Island, NY, USA), 100 nM dexamethasone, 1 µM rosiglitazone). Osteogenesis was demonstrated by alizarin red S staining of calcium deposits, while adipogenesis was demonstrated using oil red O staining of lipids. For chondrogenic differentiation, PDL cells (4 × 10^5^ cells in 20 µL of CCM) were placed into 12-well plates and incubated at 37 °C with 5% CO_2_ for 3 h attachment period to create micromass cultures. After gently adding additional CCM, the micromass cultures were rested for an additional 24 h. Differentiation was carried out by adding chondrogenic medium (CCM with 50 µg/mL L-ascorbic acid 2-phosphate, 100 nM dexamethasone, 1× insulin-transferrin-selenium premix and 10 ng/mL TGF-β3; Peprotech, Cranbury, NJ, USA). Micromasses were harvested after 3 weeks of differentiation, rinsed twice with PBS and fixed with 4% formaldehyde solution for 24 h. Micromasses were embedded in paraffin and sectioned as 5 µm thick slices. The paraffin sections were then deparaffinized in xylene, rehydrated and stained with alcian blue to visualize proteoglycans. The specificity of the detection procedures used to visualize differentiated cells was verified using non-differentiated cells as controls.

### 4.3. Flow Cytometry Analysis

Flow cytometry analysis of single-cell suspensions of PDL cells and cultured PDL cells was performed with fluorochrome-conjugated mouse monoclonal antibodies for the detection of cell surface markers (BD Biosciences, Franklin Lakes, NJ, USA). Hematopoietic cells were identified as cells expressing CD45. Mesenchymal cells were identified as stromal cells expressing the mesenchymal cell markers endoglin (CD105) and CD29 [26]. The gating strategy of flow cytometry analysis was as follows: the region of interest (ROI) was placed on a size/structure dot-plot (FSC/SSC) to eliminate debris and residues from dissociation. Within this ROI, doublets were excluded by size and then by structure. The viability of this population was then assessed by excluding 7-AAD-positive cells. From the pool of viable cells, CD45 cells were then discriminated. Finally, the expression of CD105 and CD29 markers was assessed on CD45^neg^ cells. Analysis was performed using a BD FACS Canto II, and results were analyzed with FACSDiva software v. 6.1.3 (BD Biosciences, Franklin Lakes, NJ, USA).

### 4.4. HSV-1 Infection Assays

The HSV-1 isolate used in this study was a clinical strain collected from the oral cavity of a healthy individual. It was initially propagated and characterized on Vero cells, a highly permissive kidney epithelial cell lineage from the African green monkey. HSV-1 viral stock was then produced in α-MEM medium using PDL cells as amplifying cells. The viral titer of the stock was determined by testing serial viral dilutions to define the 50% tissue culture infectious dose (TCID50) that promoted a cytopathic effect on PDL cells [30]. The multiplicity of infection (MOI) was calculated using the Reed and Muench method.

HSV-1 infection of PDL cells: In total, 6 different batches of PDL cells (from different donors) were used for HSV-1 infection and analyzed with IF staining and RT-PCRs. Of note, the cultured cells were initially characterized by IF vimentin staining, confirming their fibroblastic nature (Figure S1), which provided the basis for their use in subsequent infection experiments. Sub-confluent monolayers of PDL cells, cultured either in plastic wells (for RNA extraction) or on treated-glass slides (for IF staining), were infected with HSV-1 at different MOIs. At 1 h, 6 h, or 24 h p.i., PDL cells were rinsed twice with α-MEM medium before RNA extraction or IF staining. HSV-1 infections were also performed in the presence of 1 and 10 µg/mL of peptidoglycan (PEG) from *Micrococcus luteus* (Sigma-Aldrich, St. Louis, MO, USA).

HSV-1 infection of PDL fragments: PDL fragments were infected by direct incubation in a cell-free virus suspension (4 × 10^7^ TCID50/mL) for 1 h at 37 °C. Subsequently, PDL fragments were washed twice by centrifugation in α-MEM medium and incubated for an additional 3 h at 37 °C in 1 mL of α-MEM medium before the tissues were lysed for RNA extraction (RNeasy Mini kit^®^, Qiagen, Hilden, Germany).

### 4.5. Immunofluorescent Staining

PDL cells were seeded in CCM on type I collagen-coated coverslips at 50% confluency for 2 days. Adherent PDL cells were infected with HSV-1 at MOI 100 for 1 h and 24 h, then rinsed twice with 1× PBS at room temperature and fixed in 3.7% paraformaldehyde/PBS for 15 min. The cells were then quenched in 50 mM NH_4_Cl/PBS for 30 min and permeabilized for 4 min in 0.1% Triton X-100/PBS. After two 5 min washes in PBS, cells were incubated for 60 min in a 1% bovine serum albumin (BSA)/PBS solution to block non-specific antibody binding. The incubation with mouse primary antibodies (diluted 1:200 in 1% BSA/PBS) was carried out overnight at 4 °C. Mouse primary antibodies were HSV-1 ICP0 (11060), ICP4 (H943) and ICP8 (10A3) from Santa Cruz Biotechnology, human vimentin (Clone V9) from Dako/Agilent (Glostrup, Denmark) and mouse IgG1 Isotype Control from ThermoFischer Scientific (Waltham, MA, USA). After three 3 min washes in PBS, cells were incubated with a fluorescent secondary antibody (diluted 1:1000 in 1% BSA/PBS) and co-stained with 2 µg/mL DAPI. The secondary antibody was an Alexa Fluor^®^ 488-conjugated donkey anti-mouse IgG H&L from Abcam (Cambridge, UK). After a 30 min incubation in the dark at room temperature, the coverslips were washed three times for 3 min in PBS, once with distilled water and mounted onto microscope slides using Fluoromount Aqueous Mounting Medium (Sigma Aldrich, L’lsle-d’Abeau Chesnes, France). Image acquisition was performed using a Zeiss microscope (Oberkochen, Germany). Unless otherwise indicated, all reagents used for differentiation and staining were from Sigma Aldrich (L’lsle-d’Abeau Chesnes, France). 

### 4.6. RNA Extraction and Reverse-Transcription PCR (RT-PCR) Analysis

PDL cells grown on plastic wells were lysed for RNA extraction according to the manufacturer’s recommendations (RNeasy Mini kit^®^, Qiagen). RNA extraction from PDL fragments was performed using a GentleMACS M Tube (Miltenyi, Bergisch Gladbach, Germany) with the RNA_01 program (for fresh tissue) and Qiagen RNeasy Mini kit^®^ (tissues protocol) according to the manufacturers’ instructions. RNA quantification was performed using a microvolume spectrophotometer (SimpliNano™, Biochrom, Holliston MA, USA).

Viral and human transcripts were detected by RT-PCR using Power SYBR^®^ Green PCR Master Mix (Applied Biosystems™, Carlsbad, CA, USA). PCR experiments were performed using QuantStudio™ 5 (Applied Biosystems™) in a 20 µL final volume using 20 ng cDNA from PDL cells and 5.6 ng cDNA from PDL fragments (equivalent RNA). Amplification conditions were as follows: 95 °C, 10 min; (95 °C, 15 s; 60 °C, 1 min) cycled 40 times. Each sample was run in triplicates using specific primer sets (Table 1) for the immediate-early (ICP0 and ICP4) and early (ICP8) HSV-1 viral transcripts, pro-inflammatory interleukin-1 beta (IL-1β), tumor necrosis factor alpha (TNF-α), type 1 interferons alpha-1 and beta-1 (IFN-α and IFN-β) and type III interferon lambda 1 (IFN-λ). Relative gene expression levels were calculated using the 2^−ΔΔCT^ method, with the GAPDH gene as the reference gene. Uninfected PDL cells were used as reference samples to normalize PDL cell gene expression, and uninfected PDL fragments from the same donors served as reference controls for comparison with HSV-1 exposed fragments.

## Figures and Tables

**Table 1:** List of primers.

	Forward Primer (5′ → 3′)	Reverse Primer (5′ → 3′)
GAPDH	GGTGGTCTCCTCTGACTTCAACA	GTTGCTGTAGCCAAATTCGTTGT
ICP0	GTCGCCTTACGTGAACAAGAC	GTCGCCATGTTTCCCGTCTG
ICP4	CGACACGGATCCACGACCC	GATCCCCCTCCCGCGCTTCGTCCG
ICP8	CGACAGTAACGCCAGAAG	GGAGACAAAGCCCAAGAC
IFN-α	AGAAGGCTCCAGCCATCTCTGT	TGCTGGTAGAGTTCGGTGCAGA
IFN-β	CTTGGATTCCTACAAAGAAGCAGC	TCCTCCTTCTGGAACTGCTGCA
IFN-λ	AACTGGGAAGGGCTGCCACATT	GGAAGACAGGAGAGCTGCAACT
IL-1β	CACGATGCACCTGTACGATCA	GTTGCTCCATATCCTGTCCCT
TNF-α	GCTGCACTTTGGAGTGATCG	GCTTGAGGGTTTGCTACAACA

## Data Availability

The original contributions presented in the study are included in the article/Supplementary Materials, further inquiries can be directed to the corresponding author/s.

## Supplementary Materials

The supporting information can be downloaded at: https://www.mdpi.com/article/10.3390/ijms25158466/s1.
