# Supplementary material for: Herpes Simplex Virus Type 1 Infection of Human Periodontal Ligament
Source: Int J Mol Sci. 2024 Aug 2;25(15):8466. doi: 10.3390/ijms25158466 (PMC11312683; doi:10.3390/ijms25158466)
Supplement: Supplementary file 1 [file ijms-25-08466-s001.zip › ijms-3084228-supplementary.pdf]

Supplementary material

for

Herpes Simplex Virus Type 1 Infection of Human Periodontal Ligament

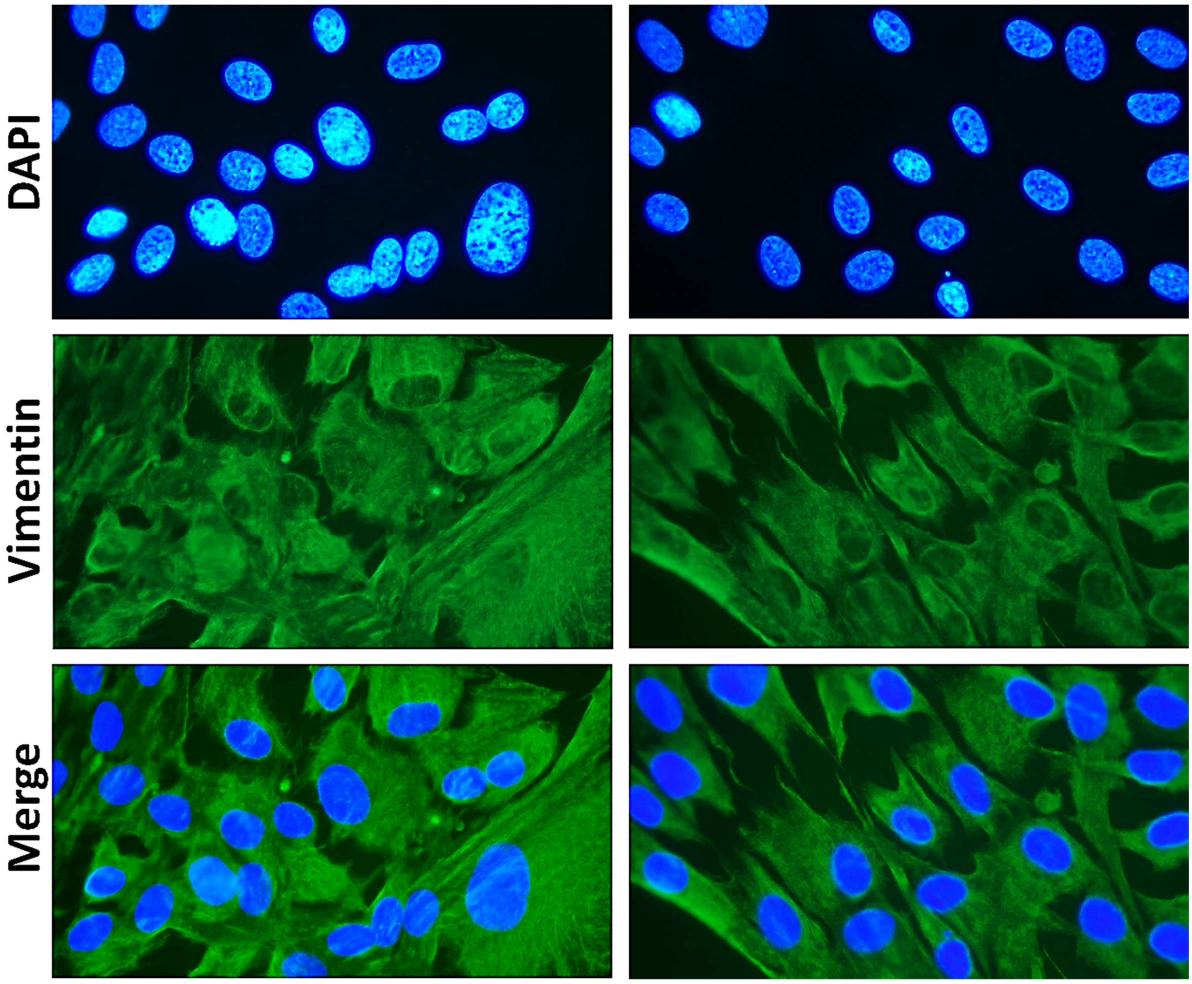

Figure S1. Immunofluorescent staining of cultured cells for vimentin, confirming their identity as fibroblasts. Cell nuclei were counterstained with DAPI. Representative micrographs from duplicate experiments are shown. Magnification, ×100.

Table S1. Statistical significance and p-values of comparisons determined using repeated measures ANOVA to delineate the viral titer-dependent (MOI) response to HSV-1 infection in PDL-cells.

	**Multiple comparisons**	**Significance**	**P-values**
**ICP0 1h**	MOI 0 vs. MOI 10	ns	0.5196
	MOI 0 vs. MOI 100	**	0.0092
	MOI 0 vs. MOI 1 000	****	<0.0001
	MOI 0 vs. MOI 10 000	****	<0.0001
	MOI 0 vs. MOI 40 000	****	<0.0001
	MOI 10 vs. MOI 100	ns	0.6975
	MOI 10 vs. MOI 1 000	**	0.0026
	MOI 10 vs. MOI 10 000	****	<0.0001
	MOI 10 vs. MOI 40 000	****	<0.0001
	MOI 100 vs. MOI 1 000	ns	0.2197
	MOI 100 vs. MOI 10 000	***	0.0004
	MOI 100 vs. MOI 40 000	****	<0.0001
	MOI 1 000 vs. MOI 10 000	ns	0.1945
	MOI 1 000 vs. MOI 40 000	**	0.0024
	MOI 10 000 vs. MOI 40 000	ns	0.7190
**ICP0 6h**	MOI 0 vs. MOI 10	***	0.0005
	MOI 0 vs. MOI 100	****	<0.0001
	MOI 0 vs. MOI 1 000	****	<0.0001
	MOI 0 vs. MOI 10 000	****	<0.0001
	MOI 0 vs. MOI 40 000	****	<0.0001
	MOI 10 vs. MOI 100	ns	0.6463
	MOI 10 vs. MOI 1 000	****	<0.0001
	MOI 10 vs. MOI 10 000	****	<0.0001
	MOI 10 vs. MOI 40 000	****	<0.0001
	MOI 100 vs. MOI 1 000	***	0.0007
	MOI 100 vs. MOI 10 000	***	0.0001
	MOI 100 vs. MOI 40 000	****	<0.0001
	MOI 1 000 vs. MOI 10 000	ns	>0.9999
	MOI 1 000 vs. MOI 40 000	ns	0.7843
	MOI 10 000 vs. MOI 40 000	ns	0.9973
**ICP4 1h**	MOI 0 vs. MOI 10	ns	>0.9999
	MOI 0 vs. MOI 100	ns	>0.9999
	MOI 0 vs. MOI 1 000	ns	0.5215

	MOI 0 vs. MOI 10 000	**	0.0034
	MOI 0 vs. MOI 40 000	***	0.0001
	MOI 10 vs. MOI 100	ns	>0.9999
	MOI 10 vs. MOI 1 000	ns	0.4784
	MOI 10 vs. MOI 10 000	**	0.0030
	MOI 10 vs. MOI 40 000	****	<0.0001
	MOI 100 vs. MOI 1 000	ns	0.6581
	MOI 100 vs. MOI 10 000	**	0.0055
	MOI 100 vs. MOI 40 000	***	0.0002
	MOI 1 000 vs. MOI 10 000	ns	0.4088
	MOI 1 000 vs. MOI 40 000	*	0.0207
	MOI 10 000 vs. MOI 40 000	ns	0.9521
**ICP4 6h**	MOI 0 vs. MOI 10	ns	0.6951
	MOI 0 vs. MOI 100	*	0.0437
	MOI 0 vs. MOI 1 000	****	<0.0001
	MOI 0 vs. MOI 10 000	****	<0.0001
	MOI 0 vs. MOI 40 000	****	<0.0001
	MOI 10 vs. MOI 100	ns	0.9264
	MOI 10 vs. MOI 1 000	**	0.0011
	MOI 10 vs. MOI 10 000	***	0.0003
	MOI 10 vs. MOI 40 000	****	<0.0001
	MOI 100 vs. MOI 1 000	*	0.0419
	MOI 100 vs. MOI 10 000	*	0.0129
	MOI 100 vs. MOI 40 000	**	0.0016
	MOI 1 000 vs. MOI 10 000	ns	>0.9999
	MOI 1 000 vs. MOI 40 000	ns	0.9681
	MOI 10 000 vs. MOI 40 000	ns	0.9997
**ICP8 1h**	MOI 0 vs. MOI 10	ns	>0.9999
	MOI 0 vs. MOI 100	ns	0.8997
	MOI 0 vs. MOI 1 000	***	0.0008
	MOI 0 vs. MOI 10 000	****	<0.0001
	MOI 0 vs. MOI 40 000	****	<0.0001
	MOI 10 vs. MOI 100	ns	0.9948
	MOI 10 vs. MOI 1 000	**	0.0025
	MOI 10 vs. MOI 10 000	****	<0.0001
	MOI 10 vs. MOI 40 000	****	<0.0001
	MOI 100 vs. MOI 1 000	*	0.0361
	MOI 100 vs. MOI 10 000	****	<0.0001

	MOI 100 vs. MOI 40 000	****	<0.0001
	MOI 1 000 vs. MOI 10 000	**	0.0023
	MOI 1 000 vs. MOI 40 000	****	<0.0001
	MOI 10 000 vs. MOI 40 000	ns	0.3484
**ICP8 6h**	MOI 0 vs. MOI 10	****	<0.0001
	MOI 0 vs. MOI 100	****	<0.0001
	MOI 0 vs. MOI 1 000	****	<0.0001
	MOI 0 vs. MOI 10 000	****	<0.0001
	MOI 0 vs. MOI 40 000	****	<0.0001
	MOI 10 vs. MOI 100	ns	0.3072
	MOI 10 vs. MOI 1 000	****	<0.0001
	MOI 10 vs. MOI 10 000	****	<0.0001
	MOI 10 vs. MOI 40 000	****	<0.0001
	MOI 100 vs. MOI 1 000	**	0.0047
	MOI 100 vs. MOI 10 000	****	<0.0001
	MOI 100 vs. MOI 40 000	****	<0.0001
	MOI 1 000 vs. MOI 10 000	*	0.0222
	MOI 1 000 vs. MOI 40 000	**	0.0018
	MOI 10 000 vs. MOI 40 000	ns	0.9971
